# Ball in the Wall: Mesenteric Fibromatosis—a Rare Case Report

**DOI:** 10.1007/s13193-020-01070-1

**Published:** 2020-04-25

**Authors:** Abhinav Deshpande, Ankita Tamhane, Y. S. Deshpande, Radhika Pagey, Meena Pangarkar

**Affiliations:** 1 National Cancer Institute,Nagpur, 25, Outer Hingna Ring Road, Mouza-Jamtha, Nagpur,, 441108 Maharashtra India; 2 Deshpande Nursing Home, Nagpur, India

**Keywords:** Mesenteric fibromatosis, GIST, Desmoid tumor of mesentery

## Abstract

**Introduction:**

Mesenteric fibromatosis-desmoid tumor of mesentery is a rare benign soft tissue tumor of mesentery. On CT, it mimics gastrointestinal stromal tumor (GIST).

**Case Report:**

A 44-year-old female with small intestinal mass, preoperatively diagnosed radiologically and pathologically as GIST.

**Conclusion:**

Mesenteric fibromatosis is a rare tumor often mistaken for GIST. Histopathology and immunohistochemistry is the key as management of both the tumors differs.

## Introduction

Mesenteric fibromatosis, also known as desmoid tumor of mesentery is a rare benign soft tissue proliferative tumor having its origin in the mesenteric tissue. It is a locally aggressive tumor. Though it lacks malignant potential, recurrences have been documented in the literature. It is important to differentiate mesenteric fibromatosis from GIST (gastrointestinal stromal tumor) as it is its closest differential on radiology as well as on histopathology. The management of both tumors is completely different. Mesenteric fibromatosis is treated by surgical resection. R0 resection minimizes the chances of local recurrence. Treatment of GIST is surgery along with Tyrosine Kinase inhibitors. Misdiagnosis can lead to hazardous therapeutic management.

We report a case of 44-year-old female presenting with small intestinal tumor preoperatively suspected as GIST.

## Case Report

**Fig. 1:**
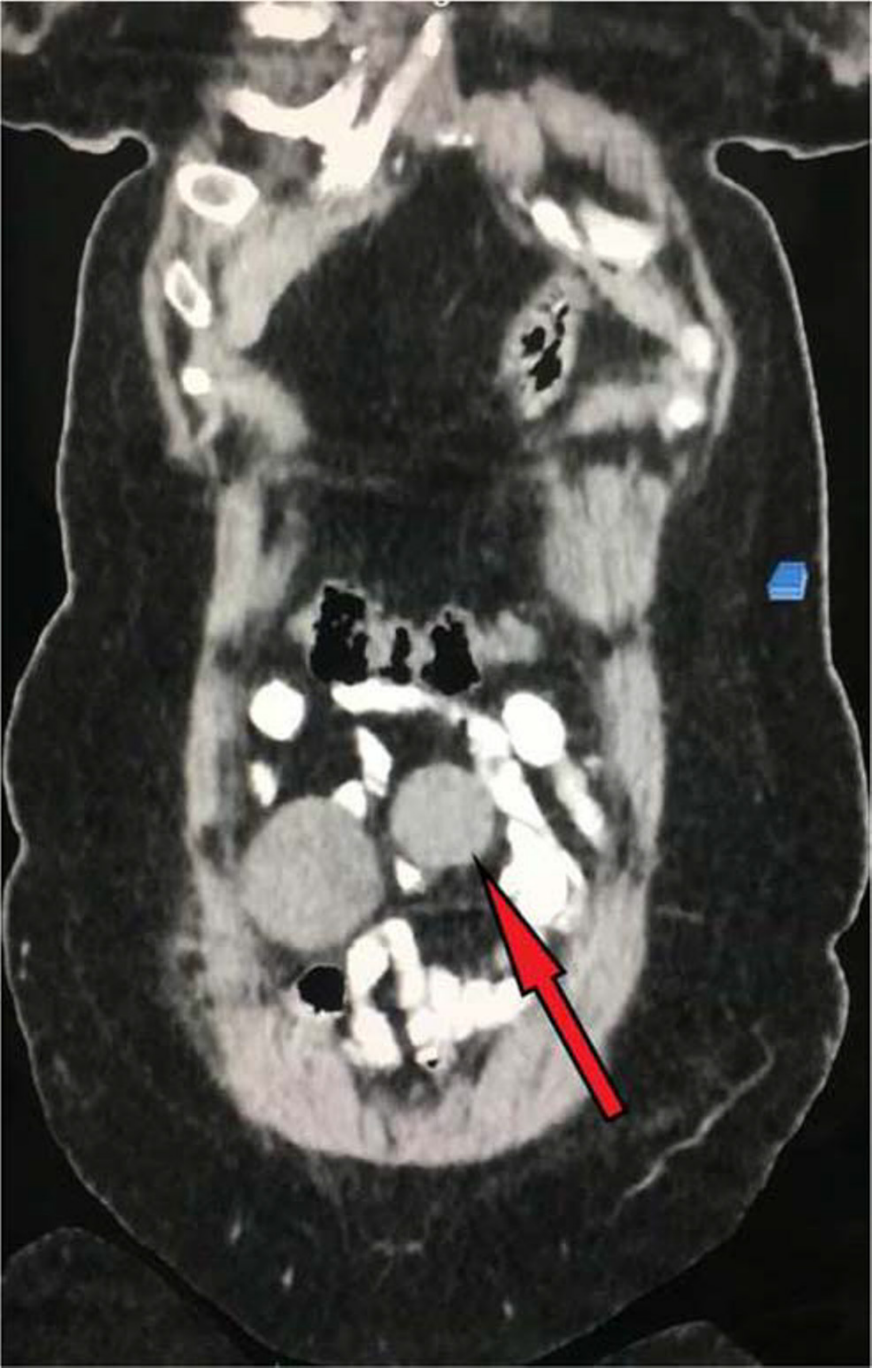
CT scan image shows a dumbbell-shaped tumor (marked by red arrow) attached to the small intestine

**Fig. 2:**
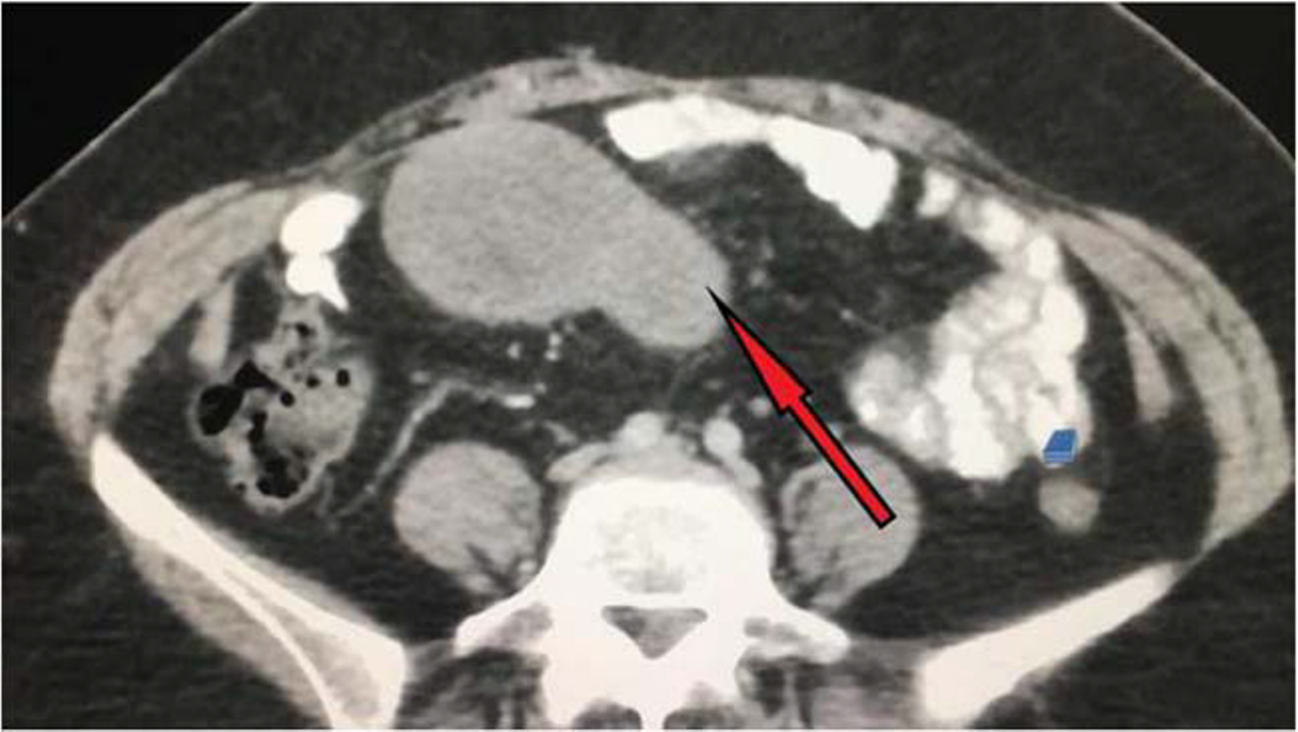
CT scan sagittal section image shows a dumbbell-shaped tumor (marked by red arrow) attached to the small intestine

A 44-year-old female presented to us with history of vague abdominal discomfort. She was previously operated for total abdominal hysterectomy for multiple fibroids and was on regular follow up. On follow up ultrasonography, the radiologist reported small intestinal soft tissue lesion and histopathological correlation was suggested. Transabdominal CT-guided biopsy was done and was reported as benign spindle cell neoplasm suspicious of GIST and was advised for immunohistochemistry (IHC) for confirmation of diagnosis. However, patient being unaffordable IHC was not performed and the patient was directly referred to us. The CECT reported the same radiological finding as that of ultrasonography revealing small intestinal soft tissue mass, well-circumscribed homogenously hypoechoic measuring approximately 11 × 7.5 × 6 cm probably originating from intestinal wall suspicious of GIST(Figs. 1 and 2).

The patient underwent exploratory laparotomy. Intraoperatively to our surprise, there were multiple soft tissue masses, largest dumbbell-shaped mass measuring approximately 11 × 7.5 × 6 cm attached to the wall of small intestine and invading the surrounding mesentery. The tumor was close to the root of mesentery; however, no gross invasion was seen. Also, there were two small nodules similar in appearance at separate sites in the same intestinal segment. For attaining R0 resection, a large segment of small intestine had to be resected measuring approximately 60 cm. Intraoperative and postoperative period was uneventful, and the specimen was sent for histopathological examination.

**Fig. 3:**
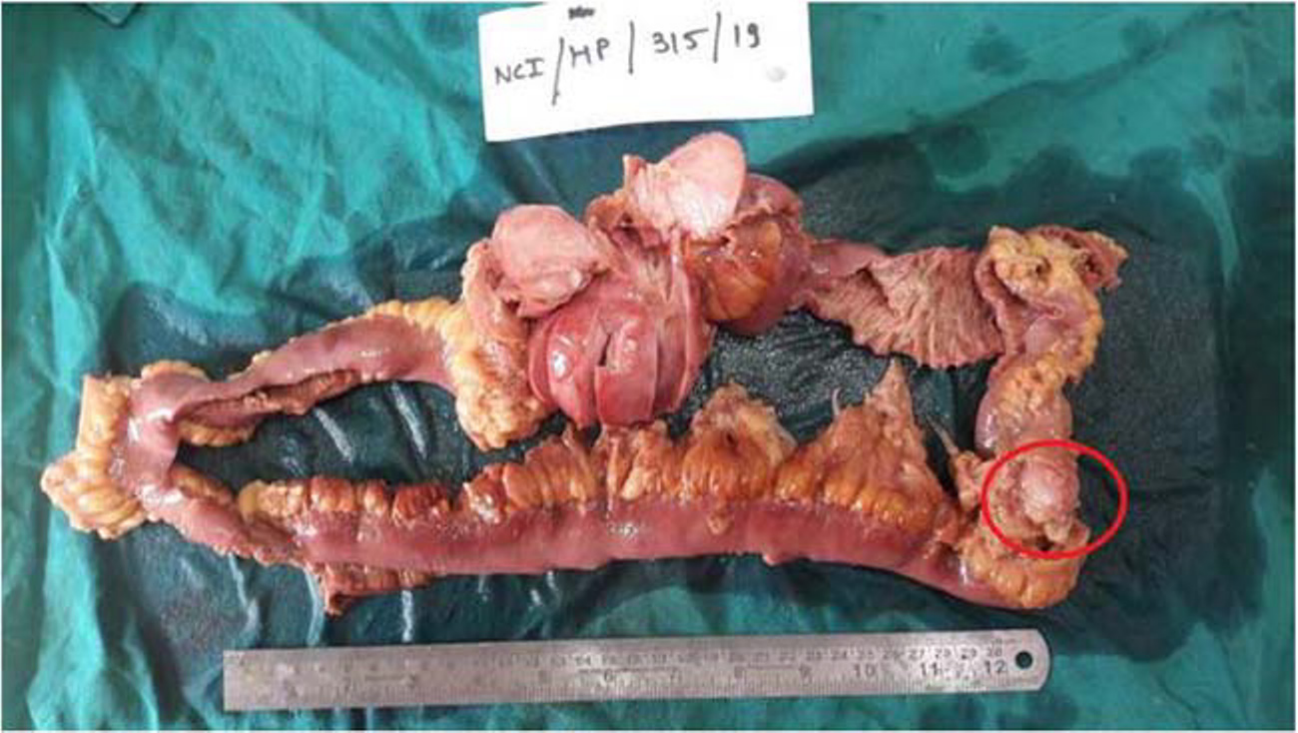
Gross of the resected specimen showing a dumbbell-shaped tumor, cut section solid white whirling seen. A smaller tumor marked by red circle is seen separately

On gross examination the intestinal segment measured approximately 60 cm in length, with a dumbbell-shaped tumor measuring approximately 11 × 7.5 × 6 cm arising from the intestinal wall and extending deep into the surrounding mesentery (Fig. 3). Another 2 smaller lesions were also identified largest measuring 1.5 × 1 × 1 cm embedded in the mesenteric tissue. The proximal resection margin was 14 cm away from the main tumor, and distal resection margin was 30 cm away from the main tumor. However, two smaller nodules closest being 8 cm from the distal resection margin were identified which were not reported on pre-operative CT. The two smaller nodules were approximately 20 cm away from the main tumor.

The tumor was well circumscribed, and the cut surface showed a solid, homogenous mass with whirling pattern without areas of hemorrhage or necrosis. Representative sections were taken for histopathological diagnosis.

**Fig. 4:**
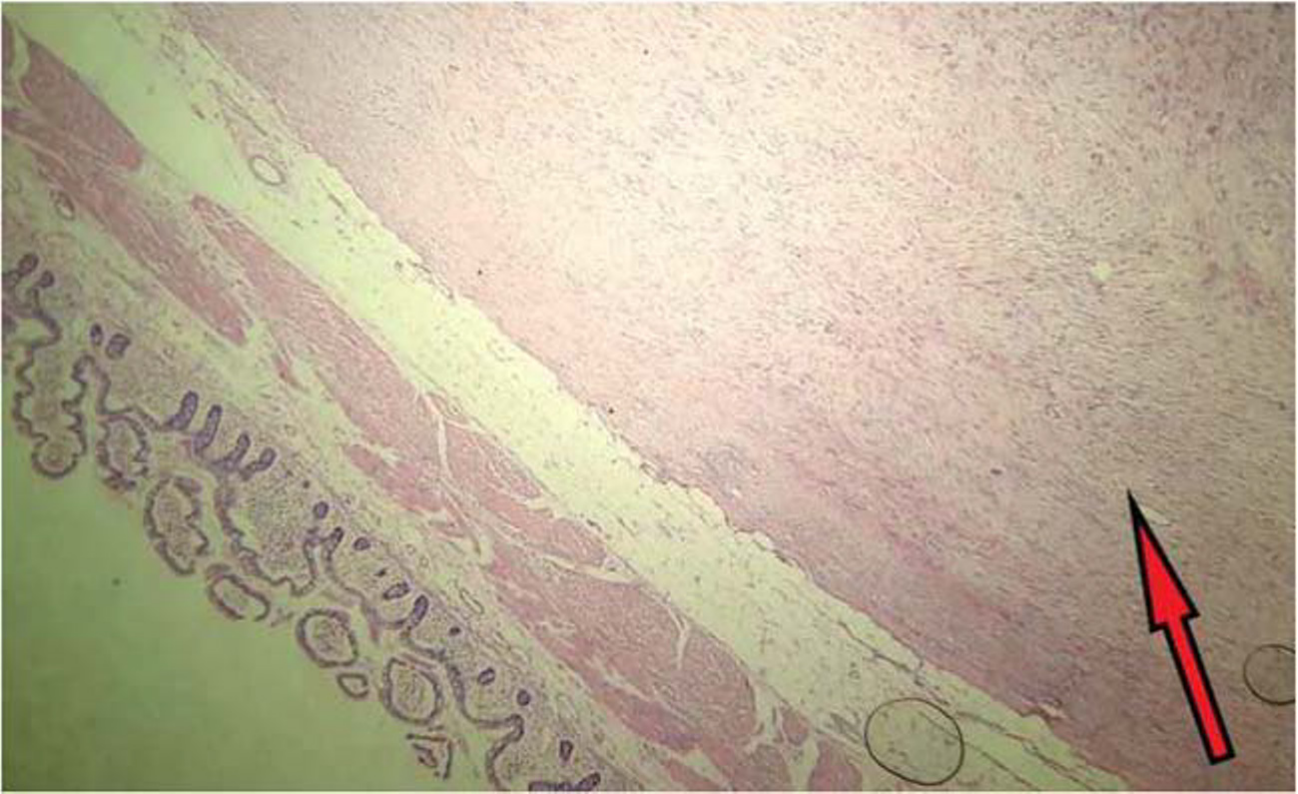
Microscopy (× 10) shows a spindle-shaped neoplasm arising from serosa. Muscularis propria and subserosa is unremarkable

**Fig. 5:**
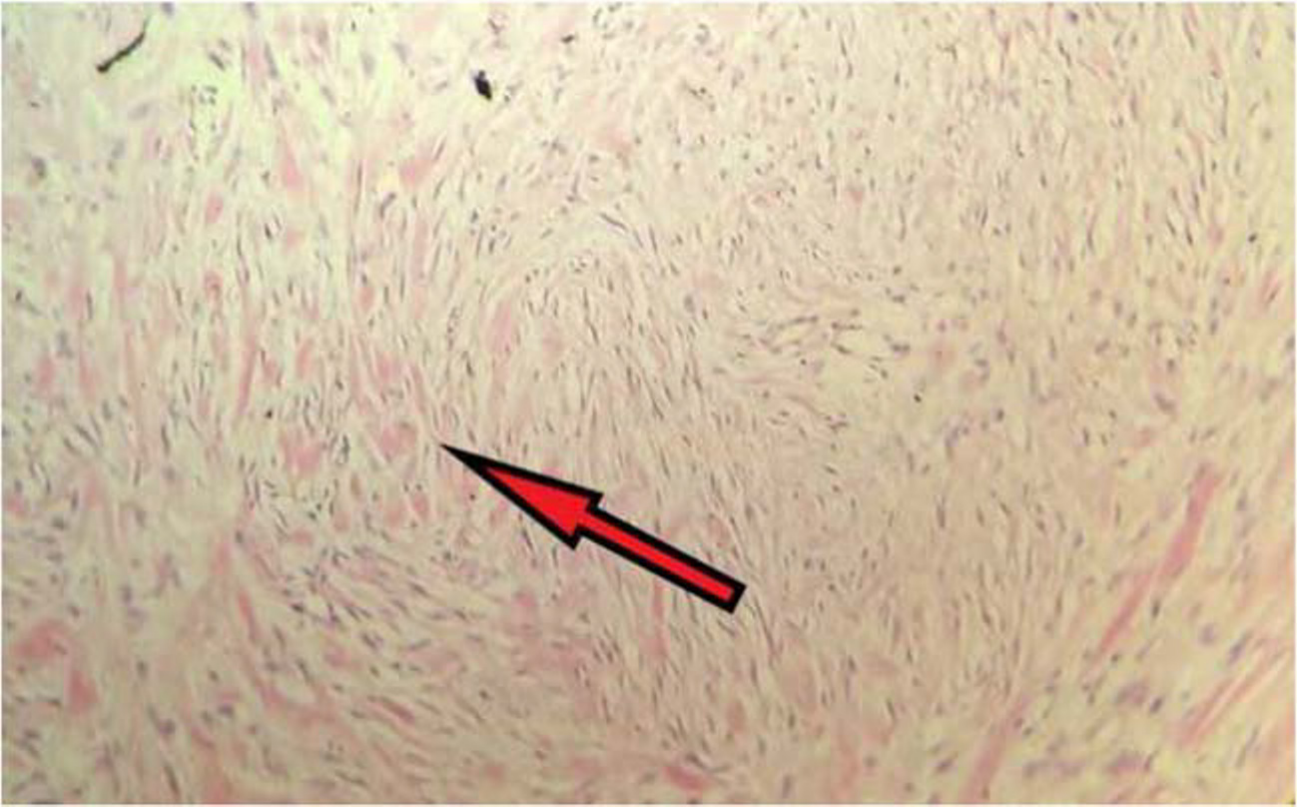
Microscopy (× 40) shows spindle cells arranged in whorls and storiform pattern. Collagenized fibers are also seen. Mitosis is not seen

On microscopy, the sections showed well-circumscribed benign spindle cell tumor arising from the serosa with cytologically bland spindle cells, stellate-shaped cells forming fascicles and storiform pattern. Prominent sclerosed blood vessels were seen with collagenized fibers. Intestinal mucosa, submucosa, muscularis propria, and subserosal fat were unremarkable (Figs. 4 and 5).

For confirmation of diagnosis, IHC was performed which showed immunoreactivity for nuclear beta catenin, vimentin, and variable SMA positivity while tumor cells were negative for CD-117, DOG-1,CD-34, and ALK-1 ruling out the differentials of GIST and inflammatory myofibroblastic tumor (IMFT). MIB 1 labeling index was 1%.

### Follow up

Immediately after 2 months of surgery, the patient presented with diarrhea because of short bowel syndrome and was managed symptomatically. The patient is post 1 year of surgery and on regular follow up with the operating surgeon and presently asymptomatic.

The patient underwent colonoscopy for evaluation of familial adenomatous polyposis (FAP); however, it was unremarkable.

## Discussion

Mesenteric fibromatosis is a benign tumor arising from the fibroblasts of mesentery. It is postulated that its occurrence may be due to previous surgical trauma, handling of the intestine and also is often related to hormonal influence. Familial adenomatous polyposis (FAP) and Gardner syndrome patients are often at risk of developing mesenteric fibromatosis.

Approximately 10% of patients develop mesenteric desmoids. FAP-associated fibromatosis have an aggressive clinical course with increased rate of recurrences. APC (adenomatous polyposis coli) gene mutations in FAP lead to overexpression of nuclear beta catenin [1–4].

Clinical course of mesenteric fibromatosis is usually uneventful after surgical resection. Rate of recurrence in sporadic cases is very low.

However, large tumors not resected for long can often cause intestinal obstruction or can lead to mesenteric ischemia. Treatment modality of mesenteric desmoids is individualized, with surgical resection done only in well-circumscribed tumors and tumors which do not invade the root of mesentery. Fifty-three to 67% of mesenteric desmoids are resectable [5].

Radiation and chemotherapy is not found useful in cases of R0 resection and patients with sporadic desmoids. Doxorubicin is given in cases of recurrent tumors and tumors with genetic mutation. Tumors which are resistant to doxorubicin have been tried with imatinib, which is most commonly useful in cases of GIST; however, its utility has not been proven in large studies (Table 1) [6].

**Table 1:** Characteristics of studies

Studies	Number of patients	Presentation	Past history	Family history	Treatment given	Outcome/follow up, recommendation
Marek Wronski et al., 2011 [7]	One (44-year-old female)	Epigastric pain	Arterial hypertension, Hashimoto thyroiditis, hypercholesterolemia History of previous cesarean section	Not significant	Elective laparotomy	No recurrence (1 year follow up)
Mukut D et al., 2014 [8]	One (29-year-old male)	Swelling on the right side of the umbilicus for 6 months and dull aching pain for 2 months	Not significant	Not significant	Exploratory laparotomy	No recurrence (3 years follow up)
Rodriguez et al., 2004 [9]	25 cases	Varied presentation depending on the organ of involvement, stomach, duodenum, ileum, jejunum, and colon. Mistaken for GIST in majority of patients.	Six patients had history of abdominal surgery in the past	Not significant in any case	Elective laparotomy	Not mentioned
Jian Li et al., 2019 [10]	One case (18-year-old female)	Acute abdomen for 10 h (abdominal pain, nausea, and vomiting)	Appendectomy 2 years back	Not significant	Exploratory laparotomy	No recurrence (17 months follow up)
Anandaravi BN et al., 2015 [11]	One case (24-year-old female)	Progressive abdominal distention associated with dull aching pain	Not significant	Not significant	Elective laparotomy	Postoperative period uneventful. Follow up not mentioned
Haibin Ji et al., 2019 [12]	One case (26-year-old male)	Abdominal distention and loss of appetite. On examination, lump in abdomen	Not significant	Not significant	Exploratory laparotomy	No recurrence (18 months follow up)
Present study, 2019	One case (44-year-old female)	Vague abdominal discomfort	Underwent total abdominal hysterectomy for multiple fibroids	Not significant	Elective laparotomy	No recurrence (1 year follow up)

## Conclusion

The present case report documents an unusual case of mesenteric fibromatosis.

IHC-proven histopathological diagnosis is mandatory for mesenteric fibromatosis as other closest differentials have a completely different surgical and therapeutic management. MRI is a preferred modality of diagnosis than CT. Usually, mesenteric fibromatosis is diagnosed as GIST on radiology and tissue diagnosis followed by IHC is mandatory.

R0 resection is vital to prevent recurrences.
